# Supplementary material for: Metabolic regulation of proteome stability via N-terminal acetylation controls male germline stem cell differentiation and reproduction
Source: Nat Commun. 2023 Oct 23;14:6737. doi: 10.1038/s41467-023-42496-9 (PMC10593830; doi:10.1038/s41467-023-42496-9)
Supplement: Supplementary file 1 — Supplementary Information [file 41467_2023_42496_MOESM1_ESM.pdf]

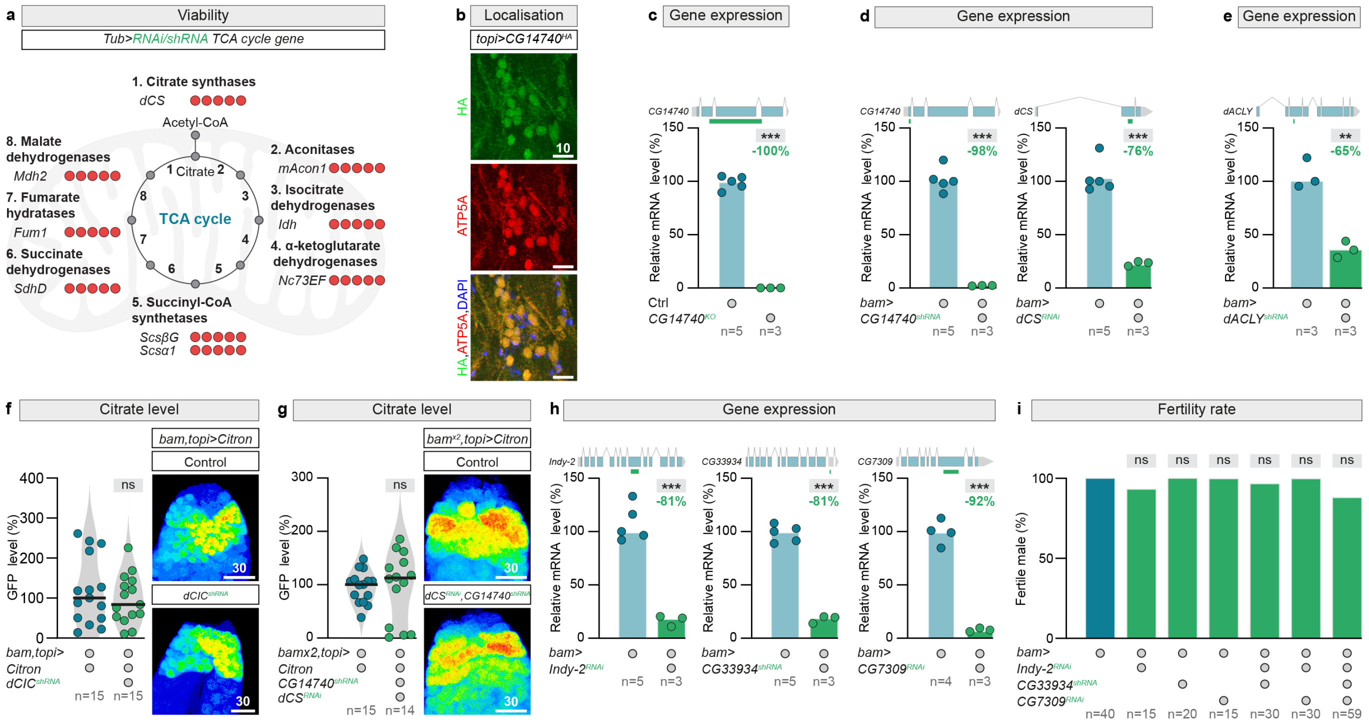

Figure S1. The RNAi lines targeting the TCA cycle genes are efficient.

a, The number of dead (red circles) or alive individuals (green circles), expressing one RNAi or shRNA targeting a specific TCA cycle gene under the control of the *tubulinP-Gal4* (*tubP-Gal4*) driver (ubiquitous driver active in all somatic cells). **b**, Representative images (DNA: DAPI, blue; HA: green, mitochondria: ATP5A, red) of CG14740^{HA} localisation at the nebenkern stage when expressed under the control of the *topi-Gal4* driver. **c**, RT-qPCR expression data for *CG14740* in dissected testes of control males and *CG14740* knock-out males. In this and all subsequent figures, expression abundance for each gene was arbitrarily set up at 100% for control males, and percentage of that expression is displayed for the other genotypes. On the top, a representation of the *CG14740* locus with the deleted region symbolised by a green line. **d**, RT-qPCR expression data for *dCS* and *CG14740* in dissected testes of control males, and males with germline-specific *CG14740* or *dCS* knock-downs. On the top, a representation of the *CG14740* and *dCS* loci with the regions targeted by the RNAi lines symbolised by a green rectangle. **e,** RT-qPCR expression data for *dACLY* in dissected testes of control males and *dACLY* knock-down males. On the top, a representation of the *dACLY* locus with the region targeted by the RNAi line symbolised by a green rectangle. **f**, Representative images and quantifications of citrate sensor signal in testes of control males and in males after germline-specific *dCIC* knock-down using *bam-Gal4* combined with *topi-Gal4*. **g**, Representative images and quantifications of citrate sensor signal in testes of control males and in males after germline-specific double *dCS* and *CG14740* knock-down using the combined *bam-Gal4* and *topi-Gal4*. **h**, RT-qPCR expression data for the three citrate transporters (*Indy-2*, *CG333934* and *CG7309*) in dissected testes of control males, and males with germline-specific citrate transporter knock-downs. On the top, a representation of the respective loci with the regions targeted by the RNAi lines symbolised by a green rectangle. **i**, Fertility of males with single, double, and triple transmembrane citrate transporter knock-downs, specifically in the male germline. Scale bars: in µm. n = number of biological replicates analysed, each replicates containing 150 testes, except in (f), (g) and (i) where n corresponds to the number of testes or flies tested.

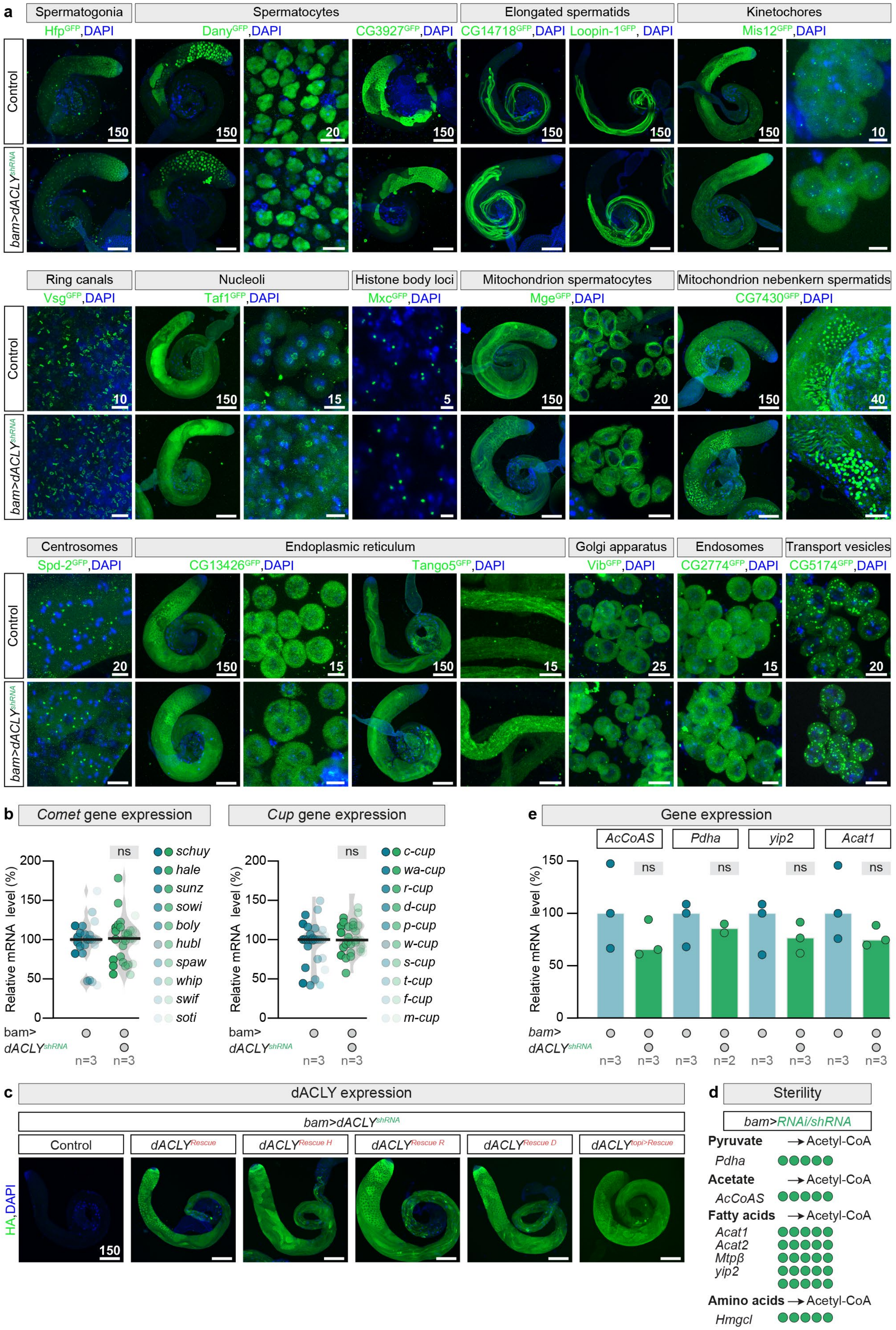
a
Spermatogonia
Spermatocytes
Elongated spermatids
Kinetochores
Hfp GFP,DAPI
Dany GFP,DAPI
CG3927 GFP,DAPI
CG14718 GFP,DAPI
Loopin-1 GFP, DAPI
Mis12 GFP,DAPI
Control
bam>dACLY shRNA
150
20
10
Ring canals
Nucleoli
Histone body loci
Mitochondrion spermatocytes
Mitochondrion nebenkern spermatids
Vsg GFP,DAPI
Taf1 GFP,DAPI
Mxc GFP,DAPI
Mge GFP,DAPI
CG7430 GFP,DAPI
15
5
40
Centrosomes
Endoplasmic reticulum
Golgi apparatus
Endosomes
Transport vesicles
Spd-2 GFP,DAPI
CG13426 GFP,DAPI
Tango5 GFP,DAPI
Vib GFP,DAPI
CG2774 GFP,DAPI
CG5174 GFP,DAPI
25
b
Comet gene expression
Cup gene expression
Relative mRNA level (%)
200
100
50
0
ns
schuy
hale
sunz
sowi
boly
hubl
spaw
whip
swif
soti
c-cup
wa-cup
r-cup
d-cup
p-cup
w-cup
s-cup
t-cup
f-cup
m-cup
bam>
dACLY shRNA
n=3
e
Gene expression
AcCoAS
Pdha
yip2
Acat1
n=2
c
dACLY expression
bam>dACLY shRNA
Control
dACLY Rescue
dACLY Rescue H
dACLY Rescue R
dACLY Rescue D
dACLY topi>Rescue
HA,DAPI
d
Sterility
bam>RNAi/shRNA
Pyruvate → Acetyl-CoA
Pdha
Acetate → Acetyl-CoA
AcCoAS
Fatty acids → Acetyl-CoA
Acat1
Acat2
Mtpβ
yip2
Amino acids → Acetyl-CoA
Hmgcl

Figure S2. Metabolic signalling through the conversion of citrate into Acetyl-CoA plays a key role in spermatid differentiation.

a, Representative images (DNA: DAPI, blue; protein, green) of Half pint (Hfp^{GFP}), Distal antenna-young ($Dany^{GFP}$), $CG3927^{GFP}$, $CG14718^{GFP}$, Loopin-1^{GFP}, $Mis12^{GFP}$, Visgun (Vsg^{GFP}), TBP-associated factor 1 ($Taf1^{GFP}$), Multi sex combs (Mxc^{GFP}), Maggie (Mge^{GFP}), $CG7430^{GFP}$, Spindle defective 2 ($Spd\text{-}2^{GFP}$), $CG13426^{GFP}$, Transport and Golgi organisation 5 ($Tango5^{GFP}$), Vibrator (Vib^{GFP}), $CG2774^{GFP}$ and $CG5174^{GFP}$ expressions in testes of control males and in males after germline-specific *dACLY* knock-down using the *bag of marbles-Gal4* (*bam-Gal4*). **b**, RT-qPCR expression data for the *Comet* (*Schuy, Hale, Sunz, Sowi, Boly, Hubl, Spaw, Whip, Swif* and *Soti*) and *Cup* genes (*C-cup, Wa-cup, R-cup, D-cup, P-cup, W-cup, S-cup, T-cup, F-cup* and *M-cup*) in dissected testes of control males, and males with germline-specific *dACLY* knock-down using *bam-Gal4*. **c,** Representative images (DNA: DAPI, blue; dACLY: HA, green) of dACLY expression in testes of control males, and males with rescued *dACLY* knock-down using the different indicated $dACLY^{HA}$ transgenes. **d**, The number of fertile (green circles) males expressing one RNAi or shRNA targeting a specific gene involved in Acetyl-CoA production under the control of *bam-Gal4* driver. **e**, RT-qPCR expression data for genes encoding the following Acetyl-CoA producing enzyme, *AcCoAS, Pdha, yip2* and *Acat1*, in dissected testes of control males, and males with germline-specific *dACLY* knock-down using *bam-Gal4*. Scale bars: in µm. n = number of biological replicates analysed, each replicates containing 150 testes.

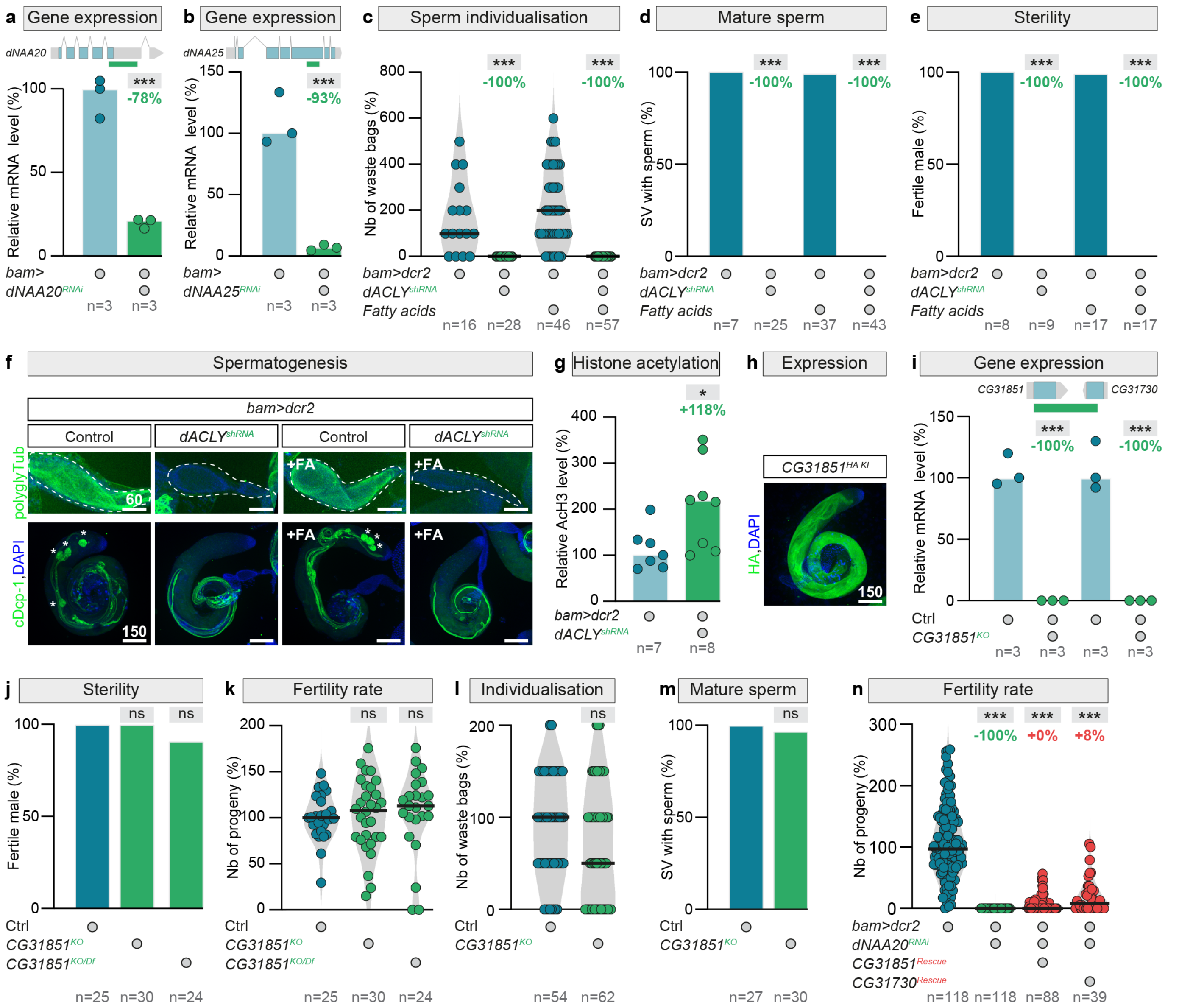

Figure S3. N-terminal protein acetylation is essential for sperm production.
a, RT-qPCR expression data for *dNAA20* in dissected testes of control males and males with *dNAA20* knock-down. On the top, a representation of the *dNAA20* locus with the region targeted by the RNAi line symbolised by a green rectangle. **b**, RT-qPCR expression data for *dNAA25* in dissected testes of control males and *dNAA25* knock-down males. On the top, a representation of the *dNAA25* locus with the region targeted by the RNAi line symbolised by a green rectangle. **c-f**, Quantifications of (**c**) the number of waste bags, (**d**) the percentage of seminal vesicles with mature sperm, (**e**) the percentage of fertile males and (**f**) representative images (DNA: DAPI, blue; protein, green) of polyglycylated α-tubulin (polyglyTub), and cleaved Dead caspase-1 (cDcp-1) expressions in testes of control males, males with germline-specific *dACLY* knock-down using *bam-Gal4* and males with rescued *dACLY* knock-down using fatty acid feeding (+FA). **g**, Western blot analysis of Histone H3 acetylation (AcH3) in total extracts of dissected testes of control males, and males with germline-specific *dACLY* knock-down using *bam-Gal4*. **h**, Representative image (DNA: DAPI, blue; CG31851: HA, green) of CG31851^{HA} knock-in expression in testes. **i**, RT-qPCR expression data for *CG31851 and CG31730* in dissected testes of control and double knock-out individuals. On the top, a representation of the *CG31851, CG31730* locus with the deleted region symbolised by a green line. **j-m**, Quantifications of (**j**) the percentage of fertile males, (**k**) the number of progenies, (**l**) the number of waste bags, and (**m**) the percentage of seminal vesicles with mature sperm in testes of control males, and double *CG31851, CG31730* knock-out males. **n**, Quantifications of the number of progenies of control males, males with germline-specific *dNAA20* knock-down using *bam-Gal4* and males with rescued *dNAA20* knock-down using *UAS-CG31851* or *UAS-CG31730* transgenes. Scale bars: in µm. n = number of biological replicates analysed, each replicates containing 150 testes in (a), (b), (g), and (i), n = number of flies tested in (e), (j), (k), and (n), n = number of testes analysed per genotype in (c) and (l), and n = number of seminal vesicles analysed in (d) and (m).

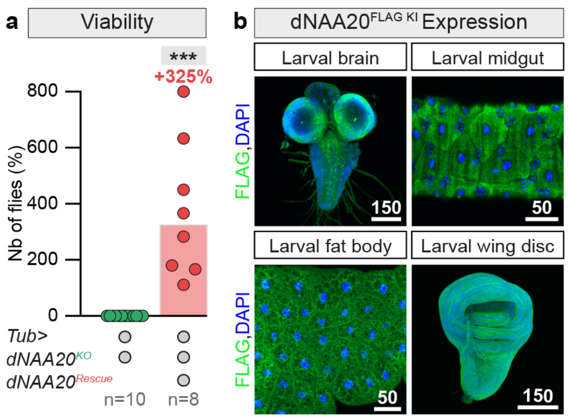

Figure S4. dNAA20 is essential for male germline differentiation.

a, Quantification of the percentage of viable *dNAA20* knock-out flies, and knock-out flies with ubiquitous re-expression of a wild-type form of dNAA20 with the *tubP-Gal4* driver. **b**, Representative image (DNA: DAPI, blue; dNAA20: FLAG, green) of dNAA20^{FLAG} knock-in expression in the indicated larval tissues. Scale bars: in µm. n = number of biological replicates analysed.

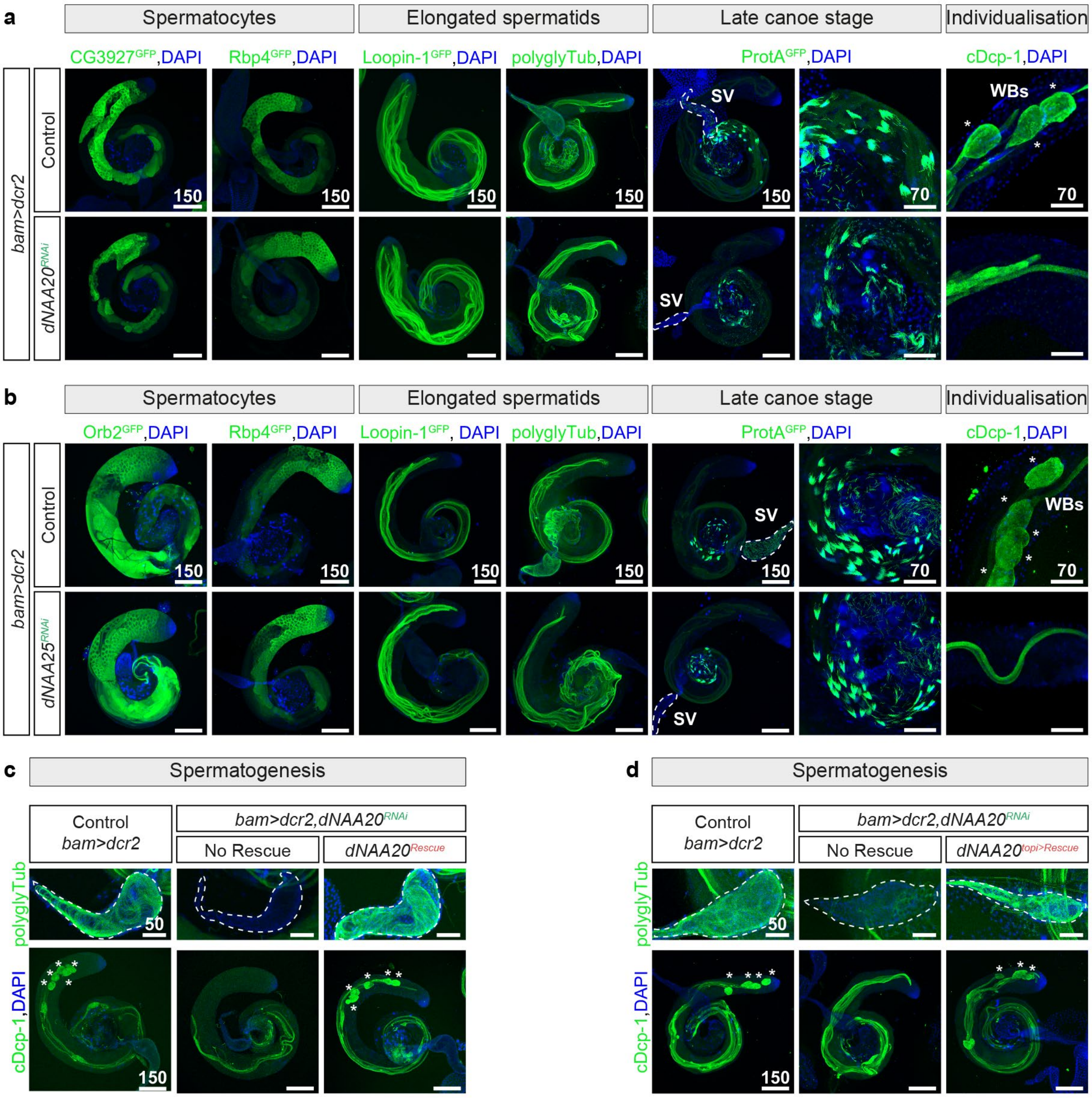

Figure S5. N-terminal protein acetylation mediated by dNAA20 is essential for spermatid differentiation.

a, Representative images (DNA: DAPI, blue; protein, green) of CG3927^{GFP}, RNA-binding protein 4 (Rbp4^{GFP}), Loopin-1^{GFP}, poly-glycylated tubulin (polyglyTub), Protamine A (ProtA^{GFP}), and cleaved Dead caspase-1 (cDcp-1) expressions in testes of control males and in males after germline-specific *dNAA20* knock-down using *bam-Gal4*. Seminal vesicles (SVs) and waste bags (WBs) are indicated by dashed lines and asterisks respectively. **b**, Representative images (DNA: DAPI, blue; protein, green) of Orb2^{GFP}, Rbp4^{GFP}, Loopin-1^{GFP}, polyglyTub, Protamine A (ProtA^{GFP}), and cDcp-1 expressions in testes of control males and in males after germline-specific *dNAA25* knock-down using *bam-Gal4*. **c**-**d**, Representative images (DNA: DAPI, blue; protein, green) of polyglyTub and cDcp-1 expressions in testes of control males, males with germline-specific *dNAA20* knock-down using *bam-Gal4* and males with rescued *dNAA20* knock-down using (**c**) a *UAS-dNAA20* or (**d**) a *topi>dNAA20* transgene.
Scale bars: in µm. See Table S4 for a list of complete genotypes.

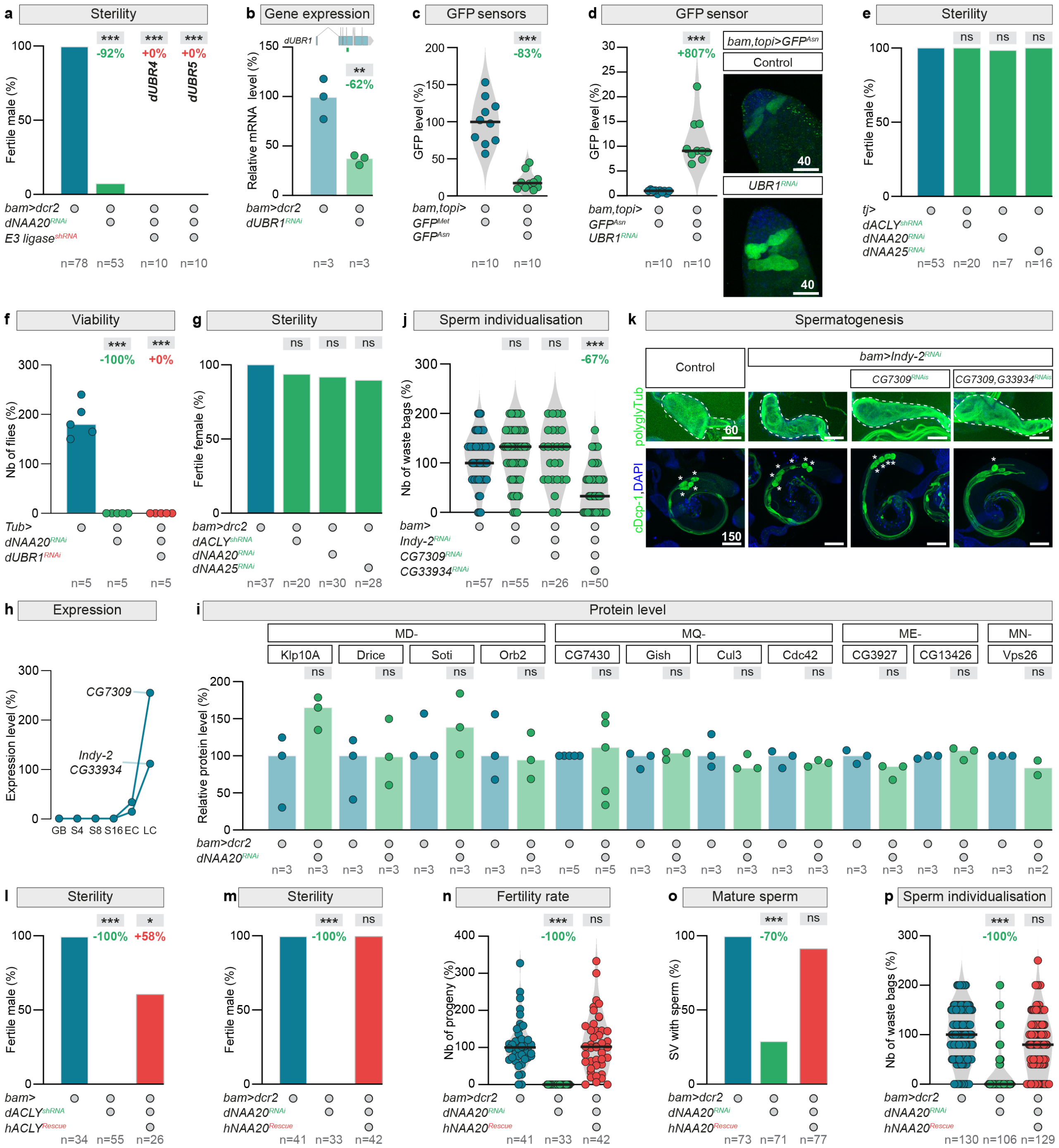

Figure S6. Context-dependent function of NatB-mediated Nt acetylation.

a, Fertility of control males, males with germline-specific *dNAA20* knock-down using *bam-Gal4* and males with double germline-specific *dNAA20* and *dUBR4* or *dUBR5* knock-downs. **b**, RT-qPCR expression data for *dUBR1* in dissected testes of control males and males with *dUBR1* knock-down. On the top, a representation of the *dUBR1* locus with the region targeted by the RNAi line symbolised by a green rectangle. **c**, Quantifications of Met-GFP and Asn-GFP reporter expressions in testes of control males. **d**, Quantifications and representative images (DNA: DAPI, blue; GFP, green) of the Asn-GFP reporter expressions in testes of control males and in males after germline-specific *dUBR1* knock-down using *bam-Gal4* combined with *topi-Gal4*. **e**, Percentage of fertile males expressing RNAis targeting *dACLY*, *dNAA20* and *dNAA25* under the control of the *traffic jam-Gal4* (*tj-Gal4*) driver (active in the somatic cells of the testes). **f**, Quantification of the percentage of viable control individuals, flies with ubiquitous somatic *dNAA20* knock-down using *tubP-Gal4*, and flies with double *dNAA20, dUBR1* ubiquitous somatic knock-downs. **g**, Percentage of fertile females expressing RNAis targeting *dACLY*, *dNAA20* and *dNAA25* under the control of the *bam-Gal4* driver (active in the female germline cells). **h**, Expression level of the three citrate transporters, *Indy2*, *CG33934* and *CG7309*, during the different spermatogenic stages (GB: gonialblasts, S4, S8, S16: cyst of 4, 8 and 16 spermatogonia; EC: early spermatocyte; LC: late spermatocyte, data adapted from[49]). **i**, Quantifications by western blot of the level of the following NatB targets: Kinesin-like protein at 10A (Klp10A), Death related ICE-like caspase (Drice), scotti (Soti), Orb2, CG7430, Gilgamesh (Gish), Cullin 3 (Cul3), Cell division cycle 42 (Cdc42), CG3927, CG13426 and Vacuolar protein sorting 26 (Vps26), in total extracts of dissected testes of control males, and males with germline-specific *dNAA20* knock-down using *bam-Gal4*. **j-k**, Quantifications of (**j**) the number of waste bags and (**k**) representative images (DNA: DAPI, blue; protein, green) of polyglycylated α-tubulin (polyglyTub), and cleaved Dead caspase-1 (cDcp-1) expressions in testes of control males, and males with germline-specific single, double, and triple transmembrane citrate transporter knock-downs. **l**, Fertility of control males, males with germline-specific *dACLY* knock-down using *bam-Gal4* and males with rescued *dACLY* knock-down using a *UAS-human ACLY* transgene. **m-p**, Quantifications of (**m**) the percentage of fertile males, (**n**) the number of progenies, (**o**) the percentage of seminal vesicles with mature sperm, and (**p**) the number of waste bags in testes of control males, males with germline-specific *dNAA20* knock-down using *bam-Gal4* and males with rescued *dNAA20* knock-down using a *UAS-human NAA20* transgene. n = number of flies tested in (a), (e), (g), (l), (m) and (n), n = number of biological replicates analysed in (b), (f), and (i), n = number of seminal vesicles analysed in (o), and n = number of testes analysed per genotype in (c), (d), (j) and (p).

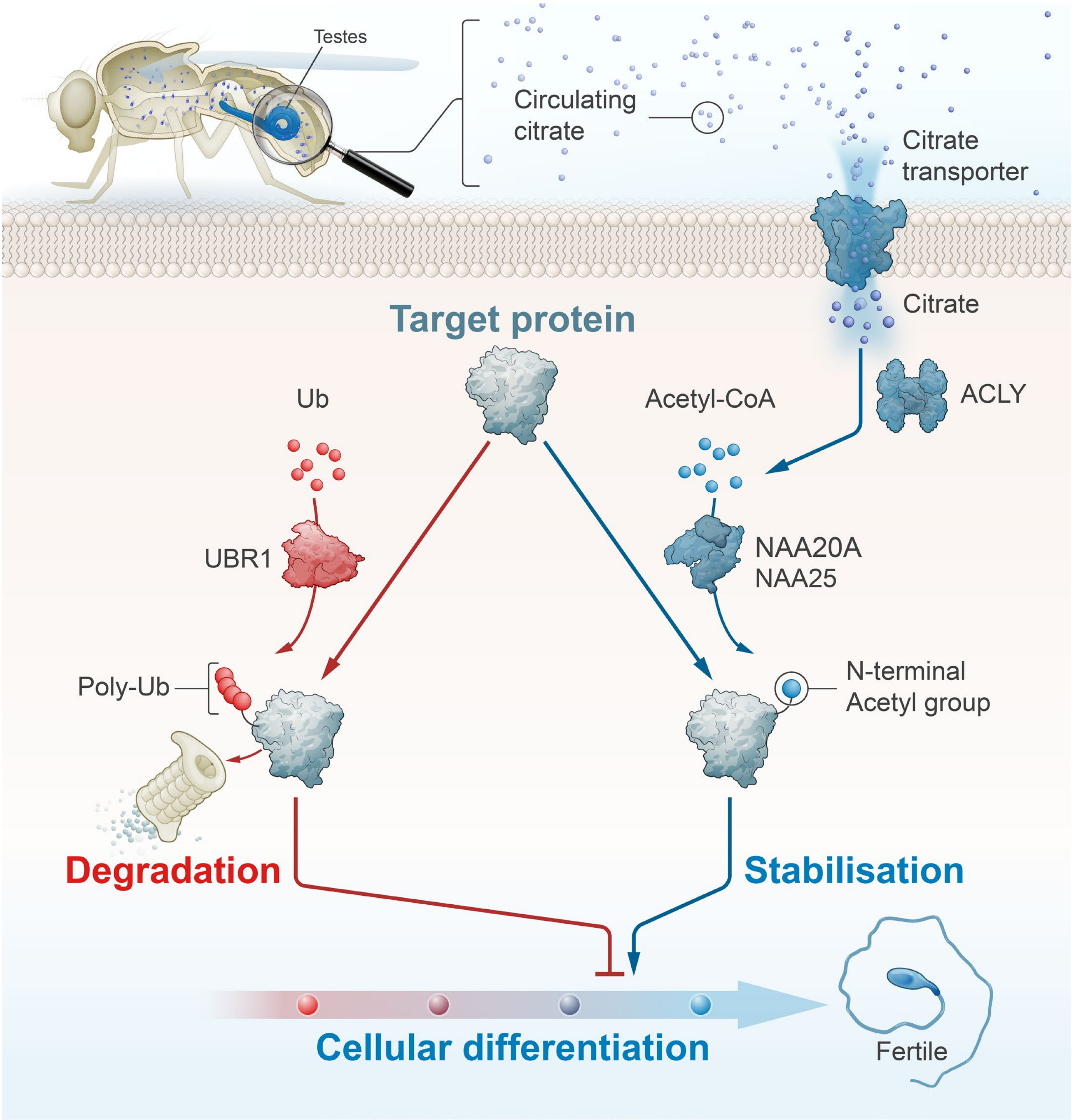

Figure S7. Metabolic regulation of proteome stability via N-terminal acetylation controls male germline stem cell differentiation.

Table S1. Fertility of males expressing an *RNAi* targeting one specific TCA cycle gene under the control of the *nanos-Gal4* driver (related to Figure 1A).

**Gene name**		**Fly line**		***nos-Gal4 x UAS-RNAi***				
				**M1**	**M2**	**M3**	**M4**	**M5**
dCS		*VDRC#107642*		F	F	F	F	F
CG14740	*CG14740*	*BL#60900*		F	F	F	F	F
mAcon1	*CG9244*	*BL#34028*		F	F	F	F	F
mAcon2	*CG4706*	*BL#58074*		F	F	F	F	F
Irp-1B	*CG6342*	*BL#67939*		F	F	F	F	F
		VDRC#110637		F	F	F	F	F
Irp-1A	*CG4900*	*BL#58117*		F	F	F	F	F
		VDRC#330238		F	F	F	F	F
Idh	*CG7176*	*BL#41708*		F	F	F	F	F
Idh3a	*CG12233*	*VDRC#106091*		F	F	F	F	F
Idh3b	*CG6439*	*BL#44475*		F	F	F	F	F
CG32026	*CG32026*	*BL#53953*		F	F	F	F	F
CG3483	*CG3483*	*VDRC#101958*		F	F	F	F	F
CG5028	*CG5028*	*VDRC#103834*		F	F	F	F	F
Nc73EF	*CG11661*	*BL#33686*		F	F	F	F	F
CG33791	*CG33791*	*BL#34101*		F	F	F	F	F
CG5214	*CG5214*	*BL#50650*		F	F	F	F	F
ScsβG	*CG10622*	*BL#50939*		F	F	F	F	F
		VDRC#101554		F	F	F	F	F
Scsα1	*CG1065*	*VDRC#107164*		F	F	F	F	F
ScsβA	*CG11963*	*BL#55168*		S	S	S	S	S
Scsα2	*CG6255*	*BL#64025*		F	F	F	F	F
SdhD	*CG10219*	*BL#65040*		F	F	F	F	F
SdhA	*CG17246*	*VDRC#330053*		F	F	F	F	S
SdhC	*CG6666*	*BL#53281*		F	F	F	F	F
		VDRC#330697		F	F	F	F	F
SdhBL	*CG7349*	*BL#58100*		F	F	F	F	F
CG6629	*CG6629*	*VDRC#106108*		F	F	F	F	F
Fum1	*CG4094*	*BL#51779*		F	F	F	F	F
		VDRC#105680		F	F	F	F	F
Fum2	*CG4095*	*VDRC#106419*		F	F	F	F	F
Fum3	*CG6140*	*BL#67379*		F	F	F	F	F
		VDRC#103522		F	F	F	F	F
Fum4	*CG31874*	*BL#65195*		F	F	F	F	F
		VDRC#103989		F	F	F	F	F
Mdh1	*CG5362*	*VDRC#110604*		F	F	F	F	F
Mdh2	*CG7998*	*BL#62230*		F	F	F	F	F
		VDRC#101551		F	F	F	F	F
CG10748	*CG10748*	*BL#62228*		F	F	F	F	F
CG10749	*CG10749*	*BL#62229*		F	F	F	F	F

Table S2. Fertility of males expressing an *RNAi* targeting one specific gene coding for an Acetyl-CoA utilising enzyme under the control of the *bam-Gal4* driver (related to Figure 3).

Gene name		Fly Line	Gal4	*X-Gal4 x UAS-RNAi*				
				M1	M2	M3	M4	M5
**Fatty acid biosynthesis**								
dACC	*CG11198*	*VDRC#8105GD*	*bam*	F	F	F	F	F
dFASN1	*CG3523*	*BL#28930*	*bam*	F	F	F	F	F
dFASN2	*CG3524*	*VDRC#105855*	*nos*	F	F	F	F	S
dFASN3	*CG17374*	*BL#63026*	*bam*	F	F	F	F	F
beg	*CG7842*	*VDRC#108556*	*bam*	F	F	F	F	S
CG12170	*CG12170*	*BL#40867*	*bam*	F	F	F	F	F
CG3603	*CG3603*	*VDRC#107046*	*bam*	F	F	F	F	S
CG16935	*CG16935*	*BL#36671*	*bam*	F	F	F	F	F
		BL#43297	*bam*	F	F	F	F	F
yip2	*CG4600*	*VDRC#26562*	*nos*	F	F	F	F	F
Mtpα	*CG4389*	*BL#32873*	*bam*	F	F	F	F	F
Echs1	*CG6543*	*BL#62221*	*bam*	F	F	F	F	F
Ppt1	*CG12108*	*BL#62291*	*bam*	F	F	F	S	S
Ppt2	*CG4851*	*BL#28362*	*bam*	F	F	F	F	F
Elo68beta	*CG11801*	*BL#50646*	*nos*	F	F	F	F	F
CG17821	*CG17821*	*BL#50898*	*nos*	F	F	F	F	F
Elo68alpha	*CG32072*	*BL#53307*	*nos*	F	F	F	F	F
CG18609	*CG18609*	*BL#44510*	*nos*	F	F	F	F	F
Mtpβ	*CG4581*	*BL#34546*	*bam*	F	F	F	F	F
**Protein acetylation**								
Hat1	*CG2051*	*BL#42488*	*bam*	F	F	F	F	F
CG1894	*CG1894*	*BL#34925*	*bam*	F	F	F	F	F
Ing3	*CG6632*	*VDRC#109799*	*nos*	F	F	F	F	F
Ing5	*CG9293*	*VDRC#102002*	*bam*	F	F	F	F	F
pont	*CG4003*	*BL#50972*	*bam*	F	F	F	F	S
Eaf6	*CG12756*	*BL#50518*	*bam*	F	F	F	F	F
e(y)3	*CG12238*	*BL#32346*	*bam*	F	F	F	F	F
d4	*CG2682*	*BL#43186*	*bam*	F	F	F	F	F
Ada2b	*CG9638*	*BL#35334*	*bam*	F	F	F	F	F
dTat	CG3967	*BL#28777*	*bam*	F	F	F	F	F
CG17003	CG17003	*VDRC#101273*	*bam*	F	F	F	F	F
dNAA20	*CG14222*	*BL#36899*	*bam*	S	S	S	S	S
		VDRC#109664	*bam*	S	S	S	S	S
dNAA25	*CG4845*	*VDRC#21960*	*bam*	S	S	S	S	S
		VDRC#103558	*bam*	S	S	S	S	S
CG31851	*CG31851*	*VDRC#104306*	*nos*	F	F	F	F	F
CG31730	*CG31730*	*VDRC#104274*	*bam*	F	F	F	F	F
		VDRC#21408	*bam*	F	F	F	F	F
		BL#42848	*bam*	F	F	F	F	F
dNAA30	*CG11412*	*VDRC#101769*	*bam*	F	F	F	F	F
dNAA35	*CG4065*	*VDRC#109595*	*bam*	F	F	F	F	F

dNAA38	*CG31950*	*VDRC#34750*	*bam*	F	F	F	F	F
CG32319	*CG32319*	*VDRC#24728*	*nos*	F	F	F	F	F
**Ketone body biosynthesis**								
CG10932	*CG10399*	*BL#51785*	*bam*	F	F	F	F	F
CG9149	*CG9149*	*BL#56858*	*bam*	F	F	F	F	F
		BL#67208	*bam*	F	F	F	F	F
Hmgs	*CG4311*	*BL#57738*	*bam*	F	F	F	F	F
Hmgcl	*CG10399*	*BL#51861*	*bam*	F	F	F	F	F
SCOT	*CG1140*	*BL#51899*	*bam*	F	F	F	F	F
sro	*CG12068*	*BL#67767*	*bam*	F	F	F	F	F
CG13377	*CG13377*	*BL#65215*	*bam*	F	F	F	F	S

Table S3. Primers used for RT-qPCR experiments

Gene	qPCR Primers		Figures
Tub	5'	*CGCGTGTGAAACACTTCCAA*	Figures 4A, S1D-F, S2B, S2E, S3B-C,S4C, S6B
	3'	*CAGCAGGCGTTTCCAATCTG*	
dNAA20	5'	*GGACATTTCGGAGAAAAAGCGA*	Figure 4A
	3'	*TGATGTAGCCAAGGTTCGTGT*	
CG14740	5'	*TAGCGTGATTGGAGGAATGCG*	Figures S1D, S1C
	3'	*CTTCGGGAGTGCCCTCTTG*	
dCS	5'	*TGCCAAATGTGGGAGCCTATG*	Figure S1D
	3'	*ATGCTGCTTGCGGAAGTTCTT*	
dACLY	5'	*GTGGAAAGCTGGGACTAATTGG*	Figure S1E
	3'	*GCGAGTAGATGCACACGTACA*	
Indy-2	5'	*GTCACGGCATTGTTTCCCCT*	Figure S1F
	3'	*TAAGCCCACCAAGGAATACCA*	
CG33934	5'	*TTCTGGATCACGGAAGCCATT*	Figure S1F
	3'	*CATAACCACGGTGTCACTAAAGT*	
CG7309	5'	*TCCTTGGTCGGTGGAAGTTTG*	Figure S1F
	3'	*GCAGCGGATATTGTGGGATTCA*	
schuy	5'	*AGTGCATAGCTGTGCTCAAAG*	Figure S2B
	3'	*GGACTTGTCATTCTTGCTCTTCT*	
hale	5'	*AGAGGGTCTGAGCATTGACTG*	Figure S2B
	3'	*CATTGAACTGGGTACAGCTCG*	
sunz	5'	*ATCTTCGACGTGACCATCATCG*	Figure S2B
	3'	*CGCTTGTATAAACCTCAAATGCG*	
sowi	5'	*TAACTTCGGAGTTGTCCCAGA*	Figure S2B
	3'	*GTCCGTTGGCCTTTGAGAAC*	
boly	5'	*TATTGACTCCCGTAAAAGATGCC*	Figure S2B
	3'	*GCTTGTAGAACATGAGGTCAGAC*	
hubl	5'	*TCCCACACGTTTCGGACAAAA*	Figure S2B
	3'	*CGGCCATACAAATAGGTGACTTG*	
spaw	5'	*TCCCACACGTTTCGGACAAAA*	Figure S2B
	3'	*CGGCCATACAAATAGGTGACTTG*	
whip	5'	*ACGAGCATCGGACAGAGATTC*	Figure S2B
	3'	*CGTCGAGGTTCGGTCTTTG*	
swif	5'	*ATGCGCGACAAGGATACCAC*	Figure S2B
	3'	*GGGATTGGTCTTGCCTCGATT*	
soti	5'	*CGCCATGCCAAAGTCAGGA*	Figure S2B
	3'	*GGCAGACCCATACCATTCGG*	
c-cup	5'	*AAGCTGGGTCCCAATTTGATG*	Figure S2B
	3'	*GGTGACAAAGTAGCCAACCAA*	
wa-cup	5'	*CCGACTGGGCCATGTTCTTTT*	Figure S2B

	3'	*CTCGGGAGCAATCCTTCAGAG*	
r-cup	5'	*GCAAATCTGAAGTCTTGGGAAAC*	Figure S2B
	3'	*CGTGCTCTCATTCTCTGTGATG*	
d-cup	5'	*GCATCGTAACCCTAATCGCAG*	Figure S2B
	3'	*CCCATGCCATTTTTGTCGTAAAC*	
p-cup	5'	*CCACGCTTTATACGGACGGAC*	Figure S2B
	3'	*GCGAGGCTCCTTTGCTTGA*	
w-cup	5'	*AAACAGACAAGATGAAAGCAGCA*	Figure S2B
	3'	*TGACCGCGCTGAACTTATTTT*	
s-cup	5'	*GGCACAGAATGTCAGATTGAACA*	Figure S2B
	3'	*CTCACCTTCATAGCGGAACTTG*	
t-cup	5'	*ATGGATGGGGAGTTAAGTGCT*	Figure S2B
	3'	*CGGGTAGATTGGTAGCCAGTA*	
f-cup	5'	*GAGAATCTGCAATCACTTACCGT*	Figure S2B
	3'	*GCGCACTAGCTTTTCCAGC*	
m-cup	5'	*ACAAAGCTCAGTCACGACCTG*	Figure S2B
	3'	*GACGAGAATCGCGGGGTAG*	
AcCoAS	5'	*CCATGATTCTGGAGCTGCCTA*	Figure S2E
	3'	*GCCTTCAGGTACAGGGGTTTC*	
Pdha	5'	*ATCATCTCGGCGTACCGTG*	Figure S2E
	3'	*GCCTCCGTAGAAGTTCGGTG*	
yip2	5'	*TCTGCCGCAACCAAAGGTATC*	Figure S2E
	3'	*GCGATCACATTTCCCACGATG*	
Acat1	5'	*ATTGCGAAGACCGATGTCCAG*	Figure S2E
	3'	*GCAGCATACATTGGTGGGC*	
dNAA20	5'	*ACCTGCGACGACCTCTTTAAG*	Figure S3B
	3'	*TGTAGAAGGACAGCCCGTAGG*	
dNAA25	5'	*GCTATGACGAAAGCCACGGAT*	Figure S3C
	3'	*GCTGGTACAGCTCCACAATTT*	
CG31851	5'	*GCACTCGGCTCTTAACAACC*	Figure S4C
	3'	*AGGACTCGTAAAGCCCAATGG*	
CG31730	5'	*GGGCAGTATCAGGTCAAGCG*	Figure S4C
	3'	*CGATGAGCATAACCCAAAGAAGT*	
dUBR1	5'	*CTTAAAGCGCGAGTCTCCCAA*	Figure S6B
	3'	*GCATAAACTCCACAACGACATCG*	

Table S4. Fly stocks used in this study

Reagent	Source	Identifier	Flybase iD	Figures
**Reporters**				
Mapmodulin^{GFP}	BDSC	51556	FBti0099819	Figure 2A
Ncd^{GFP}	BDSC	60738	FBti0167130	Figure 2A
Sm^{GFP}	BDSC	59815	FBti0178480	Figure 2A
Dj^{GFP}	BDSC	5417	FBti0013334	Figures 2A, 2E
ProtA^{GFP}	gift from B. Loppin B	N/A	FBtp0023347	Figures 2A, S5A-B
Gish^{GFP}	BDSC	59025	FBti0100581	Figure S6I
Hfp^{GFP}	VDRC	318711	FBti0198685	Figure S2A
Rbp4^{GFP}	VDRC	318563	FBti0198610	Figures S5A-B
Orb2^{GFP}	VDRC	318058	FBti0198927	Figures S5B, S6G
Loopin-1^{GFP}	gift from R. Sinka	N/A		Figures S2A, S5A-B
Dany^{GFP}	BDSC	91773	FBti0183120	Figure S2A
CG3927^{GFP}	VDRC	318780	FBti0198743	Figures S2A, S5A
CG14718^{GFP}	VDRC	318741	FBti0198842	Figure S2A
Mis12^{GFP}	BDSC	91741	FBti0214004	Figure S2A
Vsg^{GFP}	BDSC	50812	FBti0099949	Figure S2A
Taf1^{GFP}	BDSC	64451	FBti0181874	Figure S2A
Mxc^{GFP}	BDSC	84130	FBti0207696	Figure S2A
Mge^{GFP}	VDRC	318174	FBti0198764	Figure S2A
CG7430^{GFP}	VDRC	318906		Figures S2A, S6I
Spd-2^{GFP}	VDRC	318743	FBti0198658	Figure S2A
CG13426^{GFP}	VDRC	318517	FBti0198425	Figures S2A, S6I
Tango5^{GFP}	VDRC	318337	FBti0198537	Figure S2A
Vib^{GFP}	BDSC	51531	FBti0099947	Figure S2A
CG2774^{GFP}	VDRC	318605	FBti0198336	Figure S2A
CG5174^{GFP}	BDSC	50819	FBti0099757	Figure S2A
Cullin 3^{3xHA}	this study	N/A		Figure S6I
Klp10A^{GFP}	BDSC	57329	FBti0162455	Figure S6I
Cdc42^{GFP}	VDRC	318151	FBti0198614	Figure S6I
Vps26$_{GFP}$	BDSC	67153	FBti0181540	Figure S6I
**Gal4 drivers**				
bam-Gal4	gift from M. Amoyel	N/A	FBtp0111994	Figures 1, 2, 3, 5, 6, 7, S1, S2, S3, S4, S5, S6
nos-Gal4	BDSC	32563	FBtp0001612	Figures 1, 2, 3, 4, 5, 6
tj^{NP162}4-Gal4	DGGR	104055	FBti0034540	Figure S6E
TubP-Gal4	BDSC	30030	FBti0012687	Figures S1A, S4A, S6F
topi-Gal4	BDSC	91776	FBti0213638	Figure S1B, S1F-G, S6C-D
**UAS transgenes**				
UASt-CG7309	this study	N/A		Figure 1G
UASp-dACLY	this study	N/A		Figures 2B-F, 2L-N, S2C

UASp-dACLY$^{H772>A}$	this study	N/A		Figures 2L-N, S2C
UASp-dACLY$^{R380>A}$	this study	N/A		Figures 2L-N, S2C
UASp-dACLY$^{D1038>A}$	this study	N/A		Figures 2L-N, S2C
UASt-hACLY	BDSC	65837	FBti0183265	Figure S6L
UASp-dNAA25	this study	N/A		Figures 5E-I
UASt-dNAA20	this study	N/A		Figure S4A
UASp-dNAA20	this study	N/A		Figures 5A-D, 5L-N, 6D, S5C
UASp-dNAA20^{ERY}	this study	N/A		Figures 5L-M, 6E
UASp-dNAA20^{H}	this study	N/A		Figures 5L-M
UASp-dNAA20^{RG}	this study	N/A		Figures 5L-M
UASp-dNAA20^{FN}	this study	N/A		Figures 5L-M, 6F
UASp-hNAA20	this study	N/A		Figures S6M-P
UASp-CG31851	this study	N/A		Figure S3N
UASp-CG31730^{3xHA}	this study	N/A		Figure S3N
UAS-Flp	BDSC	4539	FBti0012284	Figures 4C-F, 6C
UAS-dicer2	VDRC	60010		Figures 1, 5, 6, 7, S3, S4, S5, S6
UAS-Met-GFP	gift from C. Klämbt	N/A	FBti0200441	Figure S6C
UAS-Asn-GFP	gift from C. Klämbt	N/A	FBti0200442	Figures S6C-D
UAS-Citron	this study	N/A		Figures S1F-G
UASp-CG14740^{3xHA}	this study	N/A		Figure S1B
**Mutants**				
CG31851^{KO}	this study	N/A		Figures S3I-M
CG31851$^{3xHA\ KI}$	this study	N/A		Figure S3H
dNAA20^{KO}	this study	N/A		Figures 4A, S4A
dNAA20$^{FLAG\ KI}$	this study	N/A		Figures 4B, S4B
dNAA20^{FRT}	this study	N/A		Figures 4C-F, 6C
CG14740^{KO}	this study	N/A		Figures 1B, S1C
Df(2L)BSC768	BDSC	26865	FBab0045835	Figures S3J-K
Df(3R)Exel7312	BDSC	7966	FBab0038304	Figure 1B
topi>dACLY^{3xHA}	this study	N/A		Figures 2G-I, S2C
topi>dNAA25^{3xHA}	this study	N/A		Figures 5E-I
topi>dNAA20	this study	N/A		Figures 5A-D, S5D
**RNAi transgenes**				
UAS-dCS^{RNAi}	VDRC	107642	FBti0120690	Figures 1A, S1A, S1G
UAS-dCS^{RNAi}	VDRC	26301	FBti0080130	Figures 1C, S1D
UAS-CG14740^{shRNA}	BDSC	60900	FBti0179283	Figures 1A, 1C, S1D, S1G
UAS-mAcon1^{RNAi}	BDSC	34028	FBti0140697	Figures 1A, S1A
UAS-mAcon2^{RNAi}	BDSC	58074	FBti0164392	Figures 1A
UAS-Irp-1B^{RNAi}	BDSC	67939	FBti0186731	Figures 1A
UAS-Irp-1B^{RNAi}	VDRC	110637	FBti0142187	Figures 1A
UAS-Irp-1A^{RNAi}	BDSC	58117	FBti0164459	Figures 1A
UAS-Irp-1A^{RNAi}	VDRC	330238	FBti0185955	Figures 1A
UAS-Idh^{RNAi}	BDSC	41708	FBti0149904	Figures 1A

UAS-Idh^{RNAi}	VDRC	100554	FBti0120466	Figures S1A
UAS-Idh3a^{RNAi}	VDRC	106091	FBti0120806	Figures 1A
UAS-Idh3b^{RNAi}	BDSC	44475	FBti0157339	Figures 1A
UAS-CG32026^{RNAi}	BDSC	53953	FBti0158340	Figures 1A
UAS-CG3483^{RNAi}	VDRC	101958	FBti0122320	Figures 1A
UAS-CG5028^{RNAi}	VDRC	103834	FBti0117637	Figures 1A
UAS-Nc73EF^{RNAi}	BDSC	33686	FBti0140273	Figures 1A, S1A
UAS-CG33791^{RNAi}	BDSC	34101	FBti0140705	Figures 1A
UAS-CG5214^{RNAi}	BDSC	50650	FBti0157507	Figures 1A
UAS-ScsβG^{RNAi}	BDSC	50939	FBti0158111	Figures 1A, S1A
UAS-ScsβG^{RNAi}	VDRC	101554	FBti0121565	Figures 1A
UAS-Scsα1^{RNAi}	VDRC	107164	FBti0117489	Figures 1A, S1A
UAS-ScsβA^{RNAi}	BDSC	55168	FBti0159380	Figures 1A
UAS-Scsα2^{RNAi}	BDSC	64025	FBti0180460	Figures 1A
UAS-SdhD^{RNAi}	BDSC	65040	FBti0184127	Figures 1A,S1A
UAS-SdhD^{RNAi}	VDRC	101739	FBti0121002	Figures S1A
UAS-SdhA^{RNAi}	VDRC	330053	FBti0185706	Figures 1A
UAS-SdhC^{RNAi}	BDSC	53281	FBti0157889	Figure 1A
UAS-SdhC^{RNAi}	VDRC	330697	FBti0202510	Figures 1A
UAS-SdhBL^{RNAi}	BDSC	58100	FBti0164431	Figures 1A
UAS-CG6629^{RNAi}	VDRC	106108	FBti0122779	Figures 1A
UAS-Fum1^{RNAi}	BDSC	51779	FBti0157741	Figures 1A
UAS-Fum1^{RNAi}	VDRC	105680	FBti0120862	Figures 1A, S1A
UAS-Fum2^{RNAi}	VDRC	106419	FBti0123418	Figures 1A
UAS-Fum3^{RNAi}	BDSC	67379	FBti0185631	Figures 1A
UAS-Fum3^{RNAi}	VDRC	103522	FBti0123817	Figures 1A
UAS-Fum4^{RNAi}	BDSC	65195	FBti0184282	Figures 1A
UAS-Fum4^{RNAi}	VDRC	103989	FBti0122623	Figures 1A
UAS-Mdh1^{RNAi}	VDRC	110604	FBti0142298	Figures 1A
UAS-Mdh2^{RNAi}	BDSC	62230	FBti0179012	Figures 1A, S1A
UAS-Mdh2^{RNAi}	VDRC	101551	FBti0121546	Figures 1A, S1A
UAS-CG10748^{RNAi}	BDSC	62228	FBti0179010	Figures 1A
UAS-CG10749^{RNAi}	BDSC	62229	FBti0179011	Figures 1A
UAS-dACC^{RNAi}	VDRC	8105	FBti0090448	Figure 3
UAS-dFASN1^{RNAi}	BDSC	28930	FBti0127757	Figure 3
UAS-dFASN2^{RNAi}	VDRC	105855	FBti0119829	Figure 3
UAS-dFASN3^{RNAi}	BDSC	63026	FBti0180103	Figure 3
UAS-beg^{RNAi}	VDRC	108556	FBti0116633	Figure 3
UAS-CG12170^{RNAi}	BDSC	40867	FBti0149775	Figure 3
UAS-CG3603^{RNAi}	VDRC	107046	FBti0117232	Figure 3
UAS-CG16935^{RNAi}	BDSC	36671	FBti0146682	Figure 3

UAS-CG16935^{RNAi}	BDSC	43297	FBti0151309	Figure 3
UAS-yip2^{RNAi}	BDSC	36874	FBti0146565	Figure S2D
UAS-yip2^{RNAi}	VDRC	26562	FBti0080546	Figure 3
UAS-Mtpα^{RNAi}	BDSC	32873	FBti0140375	Figure 3
UAS-Echs1^{RNAi}	BDSC	62221	FBti0179003	Figure 3
UAS-Ppt1^{RNAi}	BDSC	62291	FBti0179684	Figure 3
UAS-Ppt2^{RNAi}	BDSC	28362	FBti0127136	Figure 3
UAS-Elo68beta^{RNAi}	BDSC	50646	FBti0157502	Figure 3
UAS-CG17821^{RNAi}	BDSC	50898	FBti0157389	Figure 3
UAS-Elo68alpha^{RNAi}	BDSC	53307	FBti0157915	Figure 3
UAS-CG18609^{RNAi}	BDSC	44510	FBti0157416	Figure 3
UAS-Mtpβ^{RNAi}	BDSC	34546	FBti0140715	Figures 3, S2D
UAS-Hat1^{RNAi}	BDSC	42488	FBti0150967	Figure 3
UAS-CG1894^{RNAi}	BDSC	34925	FBti0144900	Figure 3
UAS-Ing3^{RNAi}	VDRC	109799	FBti0142087	Figure 3
UAS-Ing5^{RNAi}	VDRC	102002	FBti0121514	Figure 3
UAS-pont^{RNAi}	BDSC	50972	FBti0158154	Figure 3
UAS-Eaf6^{RNAi}	BDSC	50518	FBti0157168	Figure 3
UAS-ey3^{RNAi}	BDSC	32346	FBti0132041	Figure 3
UAS-d4^{RNAi}	BDSC	43186	FBti0150869	Figure 3
UAS-Ada2b^{RNAi}	BDSC	35334	FBti0144328	Figure 3
UAS-dTat^{RNAi}	BDSC	28777	FBti0127341	Figure 3
UAS-CG17003^{RNAi}	VDRC	101273	FBti0119276	Figure 3
UAS-dNAA20^{shRNA}	BDSC	36899	FBti0146623	Figure 3
UAS-dNAA20^{RNAi}	VDRC	109664	FBti0141766	Figures 3, 5, 6, 7, S3, S5, S6
UAS-dNAA25^{RNAi}	VDRC	21960	FBti0080678	Figures 3, 5, 6, 7, S3, S5, S6
UAS-dNAA25^{RNAi}	VDRC	103558	FBti0116761	Figure 3
UAS-CG31851^{RNAi}	VDRC	104306	FBti0120394	Figure 3
UAS-CG31730^{RNAi}	VDRC	104274	FBti0119985	Figure 3
UAS-CG31730^{RNAi}	VDRC	21408	FBti0079042	Figure 3
UAS-CG31730^{shRNA}	BDSC	42848	FBti0151179	Figure 3
UAS-dNAA30^{RNAi}	VDRC	101769	FBti0121882	Figure 3
UAS-dNAA35^{RNAi}	VDRC	109595	FBti0141310	Figure 3
UAS-dNAA38^{RNAi}	VDRC	34750	FBti0080192	Figure 3
UAS-CG32319^{RNAi}	VDRC	24728	FBti0079776	Figure 3
UAS-CG10932^{RNAi}	BDSC	51785	FBti0157747	Figure 3
UAS-CG9149^{RNAi}	BDSC	56858	FBti0163209	Figure 3
UAS-CG9149^{RNAi}	BDSC	67208	FBti0185454	Figure 3
UAS-Hmgs^{RNAi}	BDSC	57738	FBti0164187	Figure 3
UAS-Hmgcl^{RNAi}	BDSC	51861	FBti0157828	Figures 3, S2D
UAS-SCOT^{RNAi}	BDSC	51899	FBti0157866	Figure 3

UAS-sro^{RNAi}	BDSC	67767	FBti0186784	Figure 3
UAS-CG13377^{RNAi}	BDSC	65215	FBti0184141	Figure 3
UAS-dACLY^{shRNA}	BDSC	65175	FBti0184262	Figures 1, 2, S1, S2, S3, S6
UAS-dACLY^{RNAi}	VDRC	30282	FBti0090534	Figures 1D, 2J-K
UAS-CG33934^{RNAi}	VDRC	50700	FBti0087972	Figure 1E
UAS-CG33934^{RNAi}	VDRC	50699	FBti0087971	Figure 1E
UAS-CG33934^{shRNA}	BDSC	44093	FBti0158694	Figures 1G, S1H-I, S6J-K
UAS-Indy-2^{shRNA}	BDSC	34891	FBti0144864	Figure 1E
UAS-Indy-2^{RNAi}	VDRC	51048	FBti0159828	Figures 1E, 1G, S1H-I, S6J-K
UAS-Indy-2^{RNAi}	VDRC	50694	FBti0087970	Figure 1E
UAS-CG7309^{RNAi}	VDRC	100142	FBti0118895	Figures 1E, 1G, S1H-I, S6J-K
UAS-dCIC^{shRNA}	BDSC	34685	FBti0140854	Figures 1E-F, S1F
UAS-dCIC^{shRNA}	BDSC	33976	FBti0140637	Figure 1E
UAS-dUBR1^{RNAi}	BDSC	31374	FBti0130788	Figures 7A-E, 7K-L, S6B, S6D, S6F
UAS-dUBR1^{RNAi}	VDRC	108902	FBti0160098	Figures 7F-J
UAS-Pdha^{RNAi}	BDSC	55345	FBti0159564	Figure S2D
UAS-AcCoAS^{RNAi}	BDSC	41917	FBti0149942	Figure S2D
UAS-Acat1^{RNAi}	BDSC	51785	FBti0157747	Figure S2D
UAS-Acat2^{RNAi}	BDSC	56858	FBti0163209	Figure S2D
UAS-dUBR4^{shRNA}	BDSC	32945	FBti0140453	Figure S6A
UAS-dUBR5^{shRNA}	BDSC	32352	FBti0132047	Figure S6A
